# Creating the foundation data for building a population grouping methodology – lessons learned at the Canadian Institute for Health Information (CIHI)

**DOI:** 10.1186/1472-6963-15-S2-A8

**Published:** 2015-06-15

**Authors:** Heather A Richards, Jingbo Zhang, Laura Faye

**Affiliations:** 1 Case Mix Department, CIHI, Victoria, British Columbia, Canada V8W2B7; 2 Case Mix Department, CIHI, Ottawa, Ontario, Canada K2A4H6; 3 Case Mix Department, CIHI, Toronto, Ontario, Canada M2P2B7

## Background

In April 2013, CIHI initiated a project to develop a population grouping methodology that stratifies a population based on past clinical information and produces risk measures (i.e., costs for the prospective year). The methodology includes all individuals in the population at a given moment, including those who are not using the health system.

The foundation data for this project include historical clinical administrative and utilization data that are linkable at the individual level. Ideally, such a methodology includes multiple years of data that cover multiple health sectors and the full population. For example, if two years of data are used to develop clinical profiles, and if predictive indicators aim to estimate need one year in the future, then three consecutive years of data are needed. It is also important to assess the stability of the predictive indicators over time and to determine the optimal historical review period for the clinical classification; as a result, there is a need for additional years of data for such a project.

One goal for this project was for the methodology to be useful to the majority of Canadian provinces. A province will not benefit from any methodology where person-level linkable clinical information are not available to apply (vs. develop) the methodology. Clinical data only are needed to apply the final methodology, so they need to be of a consistently high quality across the provinces.

This rationale influenced the choice of health sectors to incorporate into the methodology. CIHI does not have full pan-Canadian coverage of patient-level linkable clinical data for all health sectors. Some sectors have partial or no coverage within some provinces (e.g., emergency department, home care, long-term care, drug prescriptions). Some sectors have full coverage within the provinces, but CIHI does not have access to patient-level linkable data (e.g., physician billing). Additionally, while data coverage may be sufficient in the present, historical data can be limited. These coverage issues influenced the sectors and provinces included in the foundation data.

## Materials and methods

We found that many data issues affected the methodology development. Physician billing data are critical for this methodology but, unfortunately, have notable quality issues. There are few standards or edits in place to capture diagnoses; physicians use ICD-9 to capture diagnoses but some supplement this classification with additional non-ICD codes. Physicians typically report one diagnosis per billed service, which increases the risk that comorbidities are missed. Sometimes the diagnosis reported is a symptom of an underlying disease; sometimes no diagnosis is reported for a billed service.

For the development of predictive indicators, the foundation data need to also represent the health system resource use for each individual. But, it was too restrictive to limit the foundation data to persons for whom complete cost information exists (for example, an individual might visit multiple hospitals, and/or see multiple physicians, etc. over a two-year period and all these costs need to be accounted for when building the foundation data). Instead, this project established estimation methods to address gaps in the cost data. Shadow billing was used to impute patient-level costs for the physicians on alternative payment plans. For non-case-costed hospital encounters, the cost weights generated from CIHI’s casemix methodologies were used and converted to a dollar scale.

A population grouping methodology includes health system non-users who are not currently captured by CIHI data. Initially, we believed that pseudo records for non-users could be imputed by comparing population census estimates to health system user counts in CIHI’s data. However, when compiling data over multiple years, over-coverage issues in CIHI’s data became significant: not all of those who had left the province or died could be identified with CIHI data. To address this issue, we added the registered persons database (which is maintained by provincial ministries) to the foundation data; this database tracks the enrolment start and end date for each person who is eligible to receive public health care.

## Results

Person-level cost information is a combination of actual and imputed patient-level costs. The foundation data include four Canadian provinces that have sufficient historical clinical and cost data and where a registered persons file could be obtained. The data include six consecutive years of physician claims data, hospital inpatient data, day surgery data, emergency department data, and long-term care assessment data. The health sectors included in the foundation data were chosen based on data coverage considerations as well as the relative contribution that the health sector makes to understanding disease burden in a population.

## Conclusions

Creating foundation data for the development of a population grouping methodology is a significant undertaking. This paper focuses on the elements that were important for methodology development. Not discussed in this paper are the technical challenges of manipulating very large databases and standardizing data across databases and over multiple years.

